# Molecular Quantity Variations in Human-Mandibular-Bone Osteoid

**DOI:** 10.1007/s00223-022-01017-4

**Published:** 2022-08-17

**Authors:** Anni Palander, Laure Fauch, Mikael J. Turunen, Hannah Dekker, Engelbert A. J. M. Schulten, Arto Koistinen, Nathalie Bravenboer, Arja Kullaa

**Affiliations:** 1 Institute of Dentistry, University of Eastern Finland, Yliopistonranta 1, Kuopio, 70210 Finland; 2 SIB Labs, University of Eastern Finland, Yliopistonranta 1, Kuopio, 70210 Finland; 3 Department of Applied Physics, University of Eastern Finland, Yliopistonranta 1, Kuopio, 70210 Finland; 4 Department of Oral and Maxillofacial Surgery/Oral Pathology, Amsterdam UMC and Academic Centre for Dentistry Amsterdam (ACTA), Vrije Universiteit Amsterdam, De Boelelaan, Amsterdam, 1117 The Netherlands; 5 Department of Clinical Chemistry, Amsterdam UMC, Vrije Universiteit Amsterdam, De Boelelaan, Amsterdam, 1117 The Netherlands; 6 Division of Endocrinology and Center for Bone Quality, Department of Internal Medicine, Leiden University Medical Center, PO Box 9500, Leiden, The Netherlands

**Keywords:** Bone autofluorescence, Bone FTIR, Bone modeling and remodeling, Collagen, Matrix mineralization

## Abstract

Osteoid is a layer of new-formed bone that is deposited on the bone border during the process of new bone formation. This deposition process is crucial for bone tissue, and flaws in it can lead to bone diseases. Certain bone diseases, i.e. medication related osteonecrosis, are overexpressed in mandibular bone. Because mandibular bone presents different properties than other bone types, the data concerning osteoid formation in other bones are inapplicable for human-mandibular bone. Previously, the molecular distribution of other bone types has been presented using Fourier-transform infrared (FTIR) spectroscopy. However, the spatial distribution of molecular components of healthy-human-mandibular-bone osteoid in relation to histologic landmarks has not been previously presented and needs to be studied in order to understand diseases that occur human-mandibular bone. This study presents for the first time the variation in molecular distribution inside healthy-human-mandibular-bone osteoid by juxtaposing FTIR data with its corresponding histologic image obtained by autofluorescence imaging of its same bone section. During new bone formation, bone-forming cells produce an osteoid constituted primarily of type I collagen. It was observed that in mandibular bone, the collagen type I increases from the osteoblast line with the distance from the osteoblasts, indicating progressive accumulation of collagen during osteoid formation. Only later inside the collagen matrix, the osteoid starts to mineralize. When the mineralization starts, the collagen accumulation diminishes whereas the collagen maturation still continues. This chemical-apposition process in healthy mandibular bone will be used in future as a reference to understand different pathologic conditions that occur in human-mandibular bone.

## Introduction

Bone remodeling is an important process that enables bone to adapt to different physiological conditions and replace tissue micro-damages [14]. During the bone-remodeling process, a homogeneously organic non-mineralized matrix of bone, called osteoid, is secreted by specific cells called osteoblasts [14]. Poor mineralization, defects in the collagen matrix or errors in osteoid apposition play a key part in the pathogenesis of diseases that occur primarily in jawbones (mandible and maxillary bone) [3]. Thus, molecular distribution analysis of mandibular bone osteoid will provide a better understanding of the remodeling process in the human jawbone and improve the understanding of these diseases. Few studies have focused on describing the molecular composition of remodeled bone and osteoid [19, 28, 29]. Unfortunately, these previous reports do not use the same bone section than in histological imaging, which makes it impossible to determine the exact histological localization of osteoid. Furthermore, the studies were conducted either on animal bone or on human iliac bone, but not on human jawbone. Because jawbones present different diseases and properties than those of other human-bone types and animal bone, the data of the previous studies are inapplicable to human-mandibular bone [26].

In this study, the molecular variations in human-mandibular osteoid and new bone inside cortical bone’s osteon are analyzed by using Fourier-transform infrared (FTIR) imaging spectroscopy combined with autofluorescence imaging developed previously by Fauch et al. [13]. The FTIR method is commonly used for analyzing the chemical composition of bone, but has not been previously combined with autofluorescence imaging. In the current study, the combination of high-resolution FTIR image and autofluorescence-color image of the same bone section enables accurate determination of the associated bone histology respective to its continuous chemical distribution. A precise relation between histological image and FTIR image is needed to characterize the quantity of components at a precise moment and position in the histological features (e.g. the components at the beginning of the osteoid formation and their evolution across the osteoid). However, the determination of the histological-feature localization in the FTIR data by staining the same section will not help with finding their localizations, because bone tissues are always displaced one to each other during the staining process. In contrast, the autofluorescence imaging does not require chemical staining. Thus, the different histological features observed in the autofluorescence images are positioned at the same location as in the corresponding FTIR images without any dislocation. Therefore, in this paper, we determine the precise molecular-quantity variations across different bone tissues by juxtaposing FTIR spectral images and their autofluorescence images respectives, enabling the visualization of the evolution process of organic-matrix accumulation, osteoid mineralization and relation between both processes.

## Materials and Methods

This study has used the biopsies of human-mandibular bone from ten healthy dental-implant rehabilitation patients in the Department of Oral and Maxillofacial Surgery, Alrijne Hospital, Leiderdorp in the Netherlands. The patients (women and men) were aged from 58 to 74 years old (mean 65.7 years ∓ 7.7) at the time of the biopsy. Patients did not have any general comorbidities, history of bisphosphonate medication, impaired bone metabolism, or systemic immunosuppressive medication. The levels of blood calcium, phosphate, parathyroid hormone, and HbA1c of the patients were within the normal range. All patients had given their written consent to participate in the study, and the work has been approved by the ethical committees (Medisch Ethische Toetsingscommissie (METc), Amsterdam UMC - location VUmc, Amsterdam, the Netherlands: 2011/220 and the Research Ethics Committee of Northern Savonia Hospital District: 754/2018.

This work was conducted according to the principles expressed in the Declaration of Helsinki.

### Sample Harvesting and Preparation

Cylindric bone biopsies of 10 × 2.5 mm were harvested with a 3.5 mm trephine burr (2.5 mm inner diameter) (Straumann®Dental Implant System, Straumann Holding AG, Basel, Switzerland) from dental-implant beds in the mandibular canine region under local anesthesia, one year or more after tooth extraction. An ejector pin was used to carefully remove the bone cylinder from the trephine drill. The bone samples were fixed and dehydrated with increasing concentrations of ethanol and embedded in Poly-methyl-methacrylate (PMMA: Merck KGaA). The embedded-undecalcified bone samples were cut in 3-μm-thick sections (three sections per biopsy) with a microtome (Reichert-Jung Polycut S). The cut sections were placed with a drop of ethanol on polished Zinc Selenide (ZnSe) optical windows of 13-mm diameter and of 2 mm of thickness (Crystran Limited) which transmits 70% of infrared light from 10,000 to 725 cm^−1^.

### Fourier-Transform Infrared Imaging

The bone sections were imaged by using a Fourier-transform infrared (FTIR) spectrometer (Agilent Cary 670) coupled with a microscope (Agilent Cary 620). The sections were measured in transmission mode by using a 15x-Cassegrain objective, an infrared radiation from a standard high-energy global middle-infrared light source and an infrared sensor, i.e. a liquid–nitrogen-cooled mercury-cadmium-telluride (MCT) focal-plane-array (FPA) detector consisted of 128 × 128 pixels of 1.1-μm^2^ size. The sensor captures two-dimensional distributions of bone-transmission spectra in the infrared-wavenumber range from 3800 to 750 cm^−1^ constituting hyperspectral images of 114-lp/mm optical resolution. The spectral resolution was 2 cm^−1^, and the sensor’s integration time was 0.050 ms. The hyperspectral images were obtained from the 128-co-added-spectrum average of the same bone area to provide high-quality FTIR data and were acquired by using Resolutions Pro software provided with the Agilent Cary 620 FTIR Microscope. For each different osteon, a mosaic of four 128 × 128 pixel hyperspectral images were acquired, forming thus an FTIR-hyperspectral image of 282 × 282 μm field of view of the measured osteon. FTIR images (FTIR-hyperspectral images) of ten different osteons per cortical-bone section were captured. These studied osteons have osteoid whose the mean thickness is 30 μm ∓ 12 μm.

### Autofluorescence Imaging

The bone sections were transferred from the FTIR-imaging device to the autofluorescence-imaging device to obtain two images (histological image and molecular-variation image) of the same bone sections. The sections on the ZnSe windows were covered with a cover slide glued with DPX (Dibutylphthalate polystyrene xylene, Sigma-Aldrich Inc.), and positioned in a light microscope (Zeiss AxioImager M2) in epi-illumination configuration. The sections were illuminated by light emitted from a xenon lamp coupled with an excitation filter (360/23 nm SemRock BrightLine®single-band bandpass filter). A monochrome camera (Hamamatsu ORCA-ER C4742-80) coupled to an interchangeable 20-nm-bandwidth-emission filter (Semrock BrightLine®single-band bandpass filter), has captured three different images in sequence corresponding to images of the bone autofluorescence at 390, 500 and 560 nm for each osteon. The exposure time for each acquired image was set at 10 s and the gain at 255. After the acquisition, a triband image of 0.3-μm-spatial resolution and 420 × 320-μm field of view was created from the three acquired images according to the method described by Fauch et al. [13]. The spatial resolution of the triband images was reduced to provide color images of 1.1-μm-spatial resolution corresponding to the spatial resolution of the FTIR-hyperspectral images. Afterward, the color images were juxtaposed to their corresponding FTIR-hyperspectral images by aligning the osteocytes and osteoblasts of both images.

### Data Processing

For all acquired spectra, the dark-noise subtraction and baseline correction with the FTIR spectral image of an empty ZnSe window were performed. Thereafter, the PMMA effect in tissue was removed as described previously [21]. Random-impulsive noise due to hot pixels in FTIR-multiband images (FTIR images) was reduced by filtering the acquired FTIR-multiband images by sliding a 3-by-3-median-filtering window across each spectral band of the FTIR-multiband image. The median filter was chosen because the random-impulsive noise can be reduced without blurring edges as does the mean filter [34].

### Spectral Analysis

The FTIR-absorption spectra were analyzed by using a custom-made Python script. The atom masses and interatomic-bond vibrations of minerals and proteins contained in the sample, raise characteristic absorption-spectral lines (subbands) that are partially overlapped, forming broad spectral bands in different regions of the infrared spectrum. To evaluate the subband intensities, the FTIR spectra were curve-fitted by Lorentzian subbands situated at the wavenumber positions determined by the second derivative of the spectra. The spectral positions (i.e. spectroscopic wavenumbers corresponding to the molecule-resonance frequencies) of different typical subbands of proteins and minerals are listed in Tables 1 and 2.

### Vibrational Spectral Analysis of Organic Components

The molecular properties of bone’s organic components is studied by using subbands and indexes defined in Tables 1A and B. It is noteworthy that in the osteoid area, the names of the cortical indexes do not directly reflect their indicated-collagen properties because the collagen is still accumulating. In this paper, the intensity variation of the subband at 1338 cm^−1^ is used to visualize collagen accumulation instead of the amide I spectra region because the absorption at 1338 cm^−1^ is specific to collagen type I whereas the amide I region contains water-absorption band. By consequence, the absorption in the amide I region cannot reflect the collagen content when the collagen amount is small or inexistent [23].

**Table 1:** FTIR-absorption-band assignments for organic components in bone cortex and osteoid

Assignments	Wavenumber at the resonance (${\text {cm}}^{-1}$)	Type of bonds
(A) FTIR-absorption-band assignments for organic components		
Amide I	1595–1720	C=O and C–N stretch
* Pyr*	1660	(peptide carbonyl group) [4, 9, 10]
* DPD*	1680	
* deH-DHLNL*	1690	
Amide II	1490–1590	$\delta$ N–H bend in plane and C–N and C–C stretch [4, 10]
Amide III	1215–1305	C–N stretch and N–H bend [4, 10]
Amide A	3185–3500	N–H and O–H stretch and N–H bend [8, 10]
Amide B	3000–3080	N–H stretch [10]
Collagen band	1338	C–H wag (Methylen in Proline side chain) [38]
(B) Organic indexes defined for bone cortex		
Collagen maturity (XRL)	$\frac{1660 \;}{1690 \;}$	Maturation od deH-DHLNL into Pyr [30]
Pyr/DPD ratio	$\frac{1660 \;}{1680 \;}$	Proportion of Pyr according to DPD [31]

*Pyr* hydroxylysyl-pyridinoline, *DPD* lysyl-pyridinoline, *deH-DHLNL* dehydro-dihydroxy-lysinonor-leucine

### Vibrational Spectral Analysis of Inorganic Components

The molecular properties of bone-inorganic components can be studied by using subbands and indexes defined for bone cortex listed in Tables 2A and B. In this study, the phosphate accumulation is determined by studying variations in the integrated values of the area under the $\nu _1$ ${\text{PO}}_4^{3-}$ spectral region rather than under the $\nu _3$ ${\text{PO}}_4^{3-}$ spectral region, because $\nu _3$ ${\text{PO}}_4^{3-}$ spectral region overlaps with absorption bands due to the proteins [1]. Variations in the mineral imperfections, such as a hydroxyl ${\text{HO}}^-$ or phosphate ${\text{PO}}_4^{3-}$ group substituted by a carbonate group ${\text{CO}}_3^{2-}$ , called A-type and B-type carbonate respectively, are studied by analyzing the subband-intensity variations at 880 cm^−1^ for determination of the amount of A-type-carbonate and variations at 1408, and 871 cm^−1^ for determination of the amount of B-type-carbonate. The study of A-type-carbonate variation in the $\nu _2$ ${\text{CO}}_3^{2-}$ region is preferred rather than in the $\nu _3$ ${\text{CO}}_3^{2-}$ region, because of protein overlap in this last region [24].

**Table 2:** FTIR-absorption-band assignments for minerals components in bone cortex and osteoid

Assignments	Wavenumber at the resonance ($cm^{-1}$)	Type of bonds
A. FTIR-absorption-band assignments for minerals		
Carbonate: $\nu _2$ ${\text{CO}}_3^{2-}$	850–870	C–O bend ($\nu _2$) [35]
Type B	~872	
Type A	~880	
Labile	~865	
Carbonate: $\nu _3$ ${\text{CO}}_3^{2-}$	1355–1485	O–C–O stretch ($\nu _3$) [24]
Type A1 doublet	1534–1545 & 1465–1459	
Type A2 doublet	1505 & 1565	
Type B doublet	1408–1423 & 1455–1459	
Labile	~1413 & 1500	
Acid phosphate $\nu _3$ ${\text{HPO}}_4^{2-}$	870–875	P–OH stretch ($\nu _3$)[32]
Acid phosphate: $\nu _2$ ${\text{HPO}}_4^{2-}$	988–1005	P–O stretch ($\nu _2$)
free	~988	
in HA	~987 & 1000	
Acid phosphate: $\nu _6$ ${\text{HPO}}_4^{2-}$	1037–1130	P–O stretch ($\nu _6$) [12, 32, 39]
free	~1076	
in HA	~1058 & ~1037 & 1,145	
in HA	~1127 & ~1110	
Acid phosphate : $\nu _5$ ${\text{HPO}}_4^{2-}$	1230	P–O–H bend ($\nu _5$) [32]
Phosphate: $\nu _1$ ${\text{PO}}_4^{3-}$	950–970	P–O stretch ($\nu _1$) [17, 36]
ACP	~950	
OCP	~955	
Phosphate in HA	~960	
Phosphate: $\nu _3$ ${\text{PO}}_4^{3-}$	980–1200	P–O stretch ($\nu _3$) [12, 17]
in well-crystallized HA	~1030, 1055, 1096, 1116	
in poorly crystallized HA	~1020, 1040	
Hydroxyl in HA	3570	P–O stretch (O–H)
B. Mineral indexes defined for bone’s cortex		
Mineral-to-matrix ratio (MM)	$\frac{\nu _3{\text{PO}}_4^{3-}}{{\text{Amide}} \, I}$	Mineral content per collagen amount [5, 31, 35]
Crystallinity (XST)	$\frac{1030 \;}{1020 \;}$	Poorly crystallized into more crystallized [39]
Mineral maturity (CM)	$\frac{1030 \;}{1110 \;}$	Non-apatite transformation into apatite [12, 39]
Acid phosphate substitution (APS)	$\frac{1130 \;}{1096 \;}$	Acid phosphate content in HA [39]

*HA* hydroxylapatite, *OCP* Octacalcium phosphate, *ACP*: amorphous calcium phosphate

## Results

### Osteoid and New Bone Structure in Cortical-Bone Osteon

**Fig. 1:**
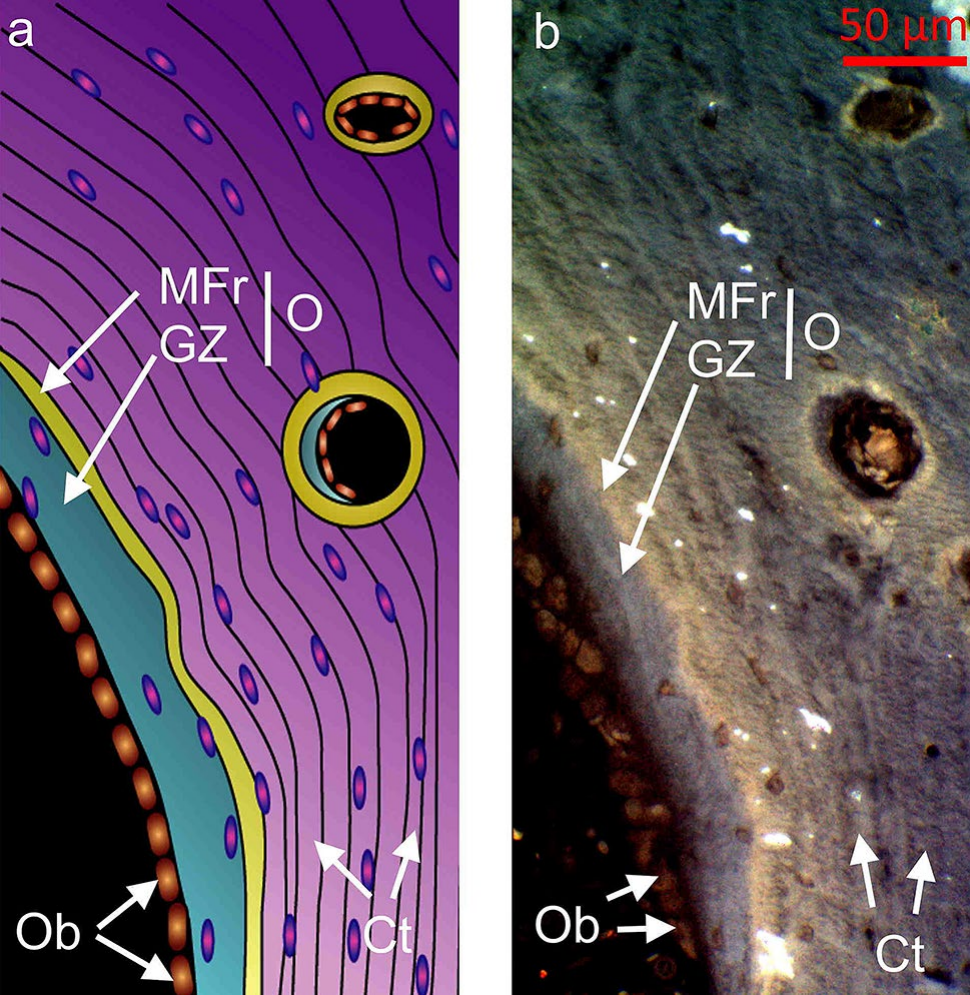
Schematic image (**a**) and autofluorescence image (**b**) of osteonal-bone osteoid and new bone with alignment of osteoblasts (Ob) adjacent to the border of osteoid, osteoid (O) consisting of a growth zone (GZ, light blue feature) and mineralizing front (MFr, bright yellow-white feature) and mineralized cortex (Ct) represented in a blue color in autofluorescence image (**b**) and in purple in schematic image (**a**)

Osteon is a bone unit that consists of lamellar cortex, surrounding the Haversian canal [15]. In the osteon, bone-forming cells (osteoblasts) align on the osteonal-bone border [14] and produce osteoid, which consists of two features: growth zone and mineralizing front [26]. A schematic representation of osteoid (O) and new bone is shown in Fig. 1a, and its corresponding autofluorescence image in Fig. 1b.

### FTIR Spectral Variations

**Fig. 2:**
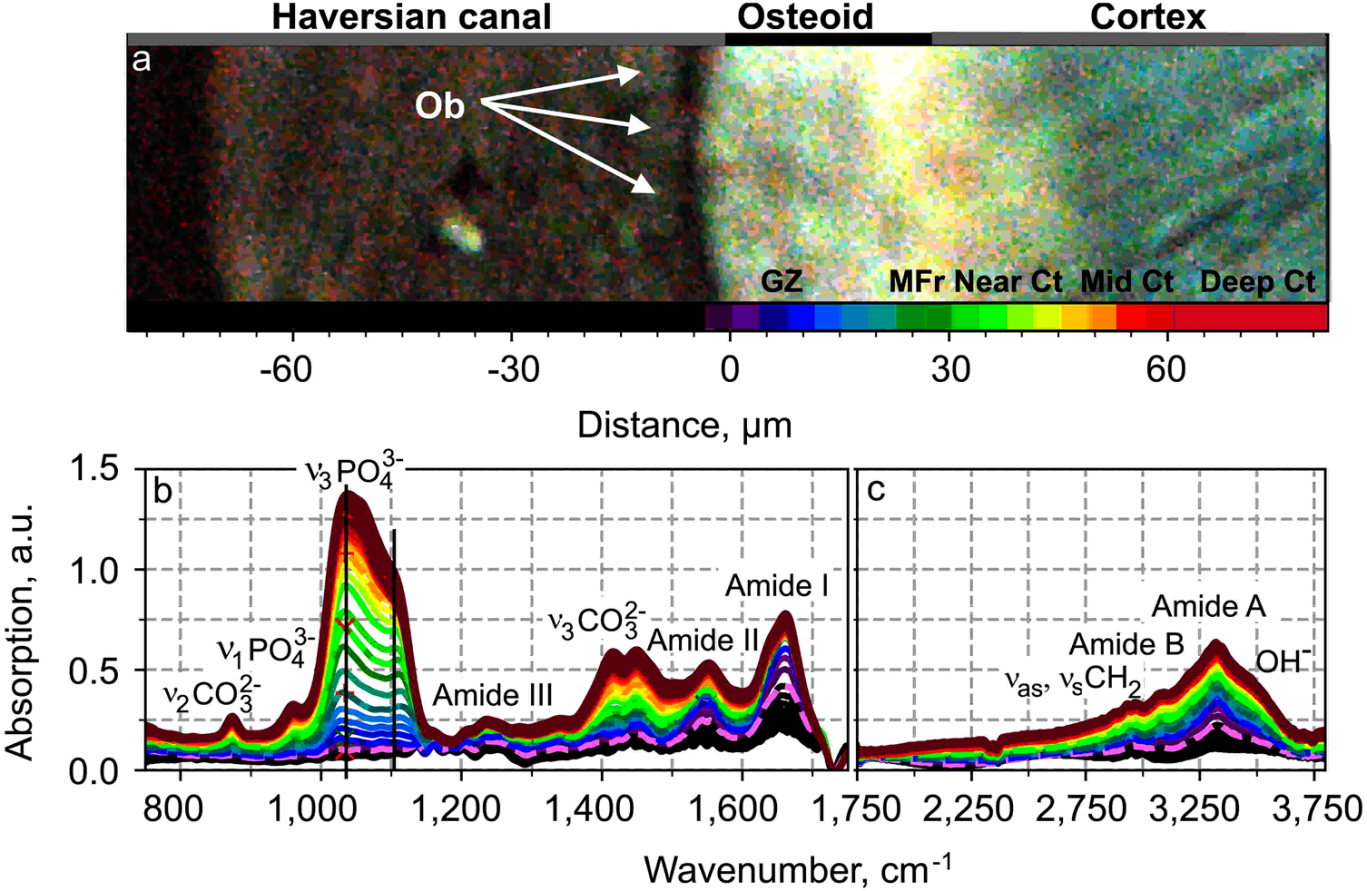
FTIR-absorption spectra of bone tissues, representative of variation spectra in all osteons of all biopsies, inside osteoid and new bone as a function of the distance from osteoblast line to deep cortex. **a** Autofluorescence image of an osteon with a color bar (from violet to dark red) indicating the anatomic locations of tissues observed in autofluorescence image, possessing the corresponding colored-FTIR spectrum presented in (**b**) and (**c**) (*Ob* osteoblasts, *GZ* growth zone, *MFr* mineralizing front, *Ct* near, middle and deep cortex)

The typical FTIR-absorption spectra of osteoid and cortical-osteonal-human-bone tissue representative of the spectra variations in all osteons of all biopsies are presented in Figs. 2b and c. They present distinct variations in magnitude and shape at different anatomic locations, represented by corresponding colors in Fig. 2a. The composition and variations between spectra are identical for each measured osteoid. Only the osteoid thickness varies from one osteoid to another, due to sample sectioning. FTIR spectra of different tissues in osteoid and new bone of osteon present absorption bands, listed previously in Table 1.

### Organic-Absorption Bands and Indexes

**Fig. 3:**
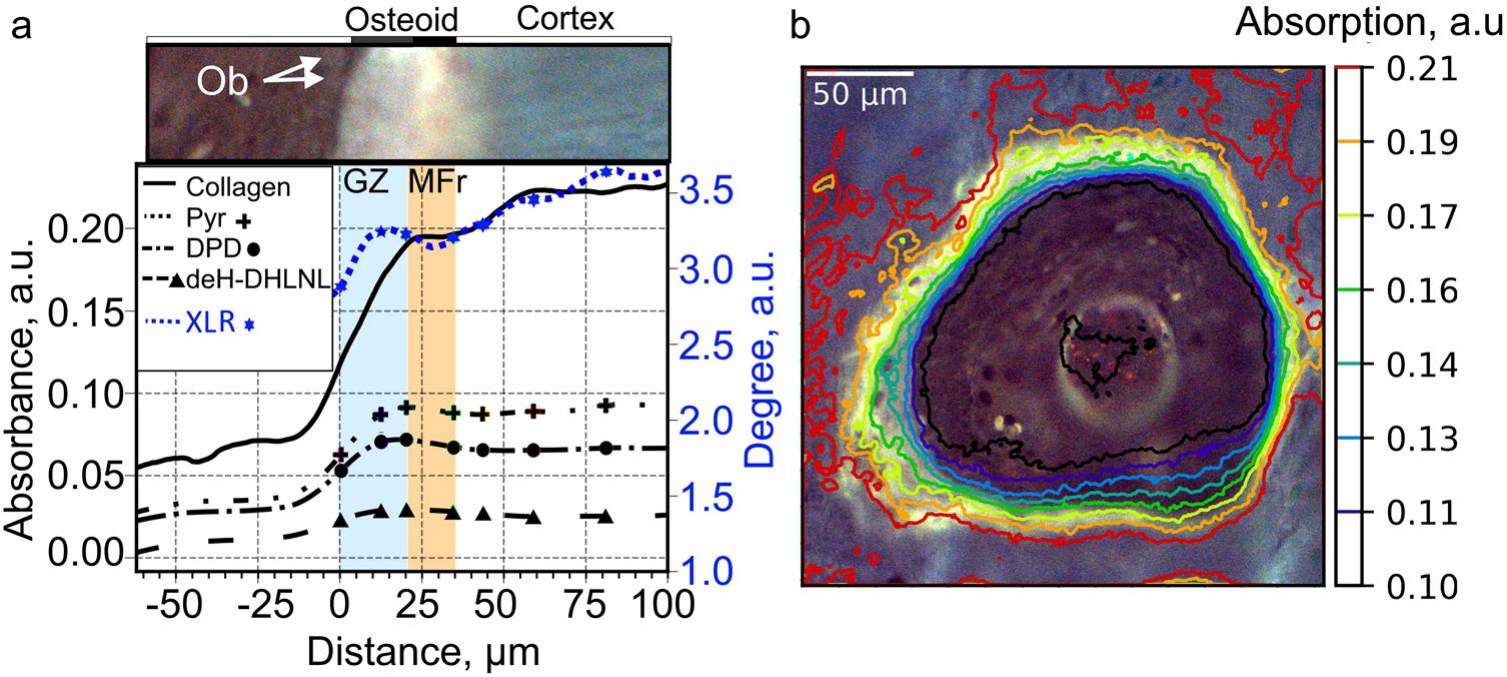
Variations in organic absorption bands and indexes representative of all osteon of all biopsies. **a** Variation of collagen-subband intensity (at 1338 cm^−1^), Pyr (at 1660 cm^−1^), deH-DHLNL (1690 cm^−1^) and DPD (1680 cm^−1^) intensity, and magnitudes of indexes: XLR (1660/1690) and Pyr/DPD (1660/1680) according to the anatomic distance from the osteoblasts. **b** Two-dimensional distribution of collagen-subband intensity (at 1338 cm^−1^) represented by FTIR equilevel lines juxtaposed on the corresponding autofluorescence color image. The equilevel lines represent the magnitude of absorption in FTIR, i.e. the amount of collagen content at each location

The spectral intensities of the absorptions in the regions amide I, amide II, amide III, amide A, and amide B increase from the osteoblast line to the end of the osteoid (at 35 μm) and beyond that, they remain almost constant. This variation indicates an organic-component accumulation inside the osteoids as shown in Fig3a and b.

As shown in Fig. 3a, the collagen-subband intensity increases linearly and strongly with the distance from the osteoblasts until the mineralizing front situated at 20 μm from osteoblast line. This strong increase is represented in Fig. 3b by the closeness of the level-lines from black to green situated in the growth zone of the osteoid. In the mineralizing front, the intensity slightly increases, indicating less collagen accumulation in this area. After the osteoid, the intensity increases anew but slowly tends to form a plateau further away in the cortex at 60 μm in Fig. 3a, i.e. at the red level-line in Fig. 3b.

In the osteoid of Fig. 3a, all subband intensities (i.e. Pyr, deH-DHLNL, and DPD subbands) increase linearly inside the growth zone (from 0 to 20 μm), whereas in the mineralizing front (from 20 to 35 μm), the subband intensities diminish. In the cortex, Pyr subband at 1660 cm^−1^ slightly increases until it reaches a plateau in deep cortex at 80  μm, DPD subband at 1680 cm^−1^ is almost constant, and deH-DHLNL subband at 1690 cm^−1^ continues to decrease in the near cortex until the middle cortex (until 45  μm) and finally slightly increase until it reaches a plateau in the deep cortex (at 80  μm). The Pyr-absorption proportion in relation to DPD absorption (Pyr/DPD-index) increases in the growth zone and cortex indicating that the increase of Pyr-absorption is more pronounced than DPD absorption and the Pyr-absorption diminution is less strong than that of DPD. In the deep cortex (after 80  μm), Pyr/DPD-index intensity reaches a plateau indicating that Pyr and DPD-absorption variations are identical in this region. The Pyr-absorption proportion in relation to deH-DHLNL absorption (XLR index) increases linearly in osteoid and less abruptly in the cortex until the deep cortex (at 80  μm) where it becomes constant, indicating that the Pyr-absorption augmentation is more pronounced than the one of deH-DHLNL. This accentuation eases in the cortex to reach identical variation in the deep cortex.

### Inorganic-Absorption Bands and Indexes

**Fig. 4:**
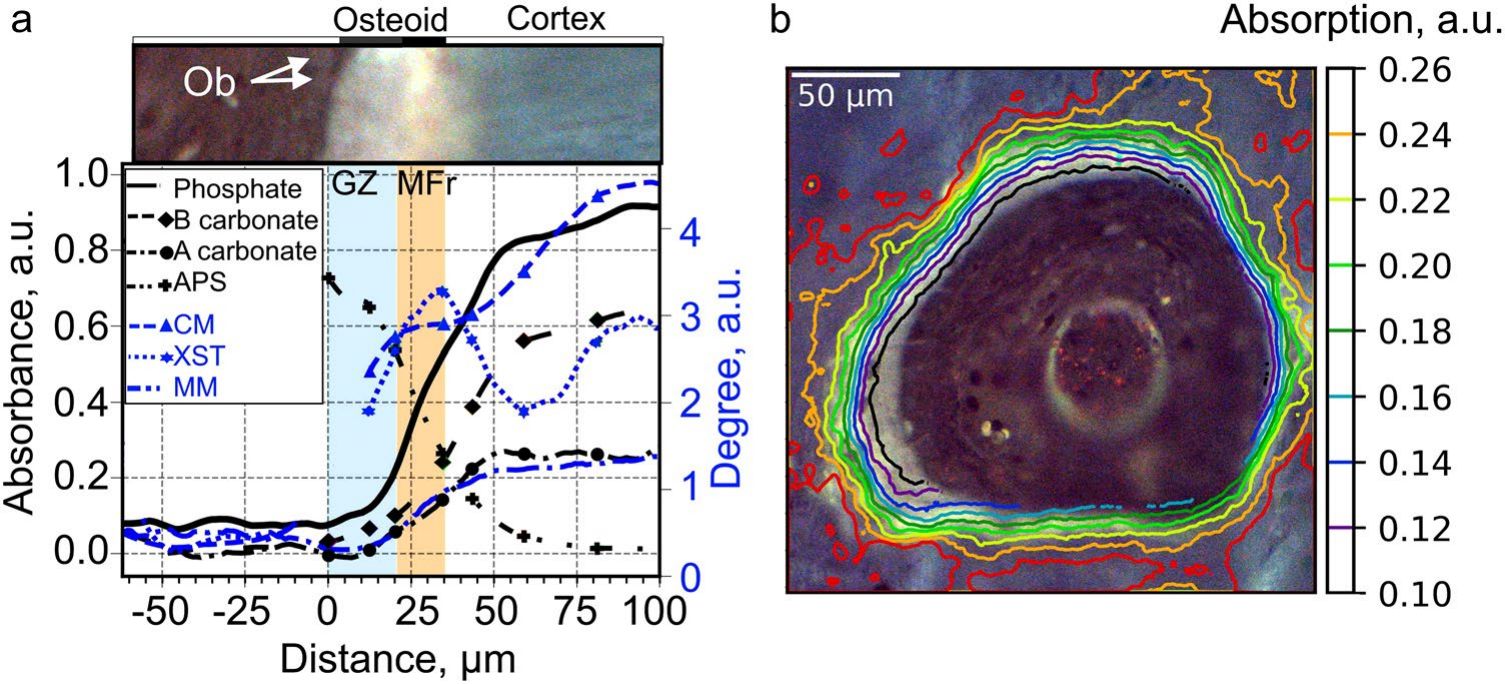
Variations in inorganic absorption bands and indexes. **a** Variation of the phosphate absorption obtained by integrating area from 950 to 970 cm^−1^ (phosphate $\nu_1$ ${\text{PO}}_4^{3-}$), of the subband at 871 cm^−1^ represents B-type carbonate’s absorption, and of the subband at 880 cm^−1^ represents A-type carbonate’s absorption and variations in magnitude of inorganic indexes: APS: 1130/1096, XST (1030/1020), CM (1030/1110) and MM according to the anatomic distance from the osteoblasts (Ob). **b** Two-dimensional distribution of phosphate-subband intensity represented by FTIR equilevel lines juxtaposed on the corresponding autofluorescence color image. The equilevel lines represent the magnitude of absorption in FTIR, i.e. the amount of phosphate content

The osteoid progressive mineralization is verified by analyzing the variation in the phosphate absorption in the $\nu_1\;{\text{PO}}_4^{3-}$ spectral region across osteoid and new bone (Fig. 4). The phosphate absorption band in the $\nu_1\;{\text{PO}}_4^{3-}$ spectral region begins to increase strongly and linearly with the distance slightly before the mineralizing front and continues to increase inside the near cortex until 55  μm in Fig. 4a. After this cut point at 55  μm, the absorption slightly increases with the distance until the deep cortex, after which, it becomes constant. This variation is also observed in Fig. 4b by level-lines appearing tight slightly before and inside the mineralizing front. The plateau is situated after the red level-line in the cortex area.

Furthermore, the spectral-absorption shape under the $\nu_1\;{\text{PO}}_4^{3-}$ spectral region changes at the interface between the growth zone and the osteoid-mineralizing front (at 20 μm). Here, a peak at around 955 cm^−1^ immerges from the noise and continues to grow with the distance in the osteoid and the nearest cortex (Fig. 2b). During its ascension (from 20 to 40 μm), the peak shifts progressively to the right from the spectroscopic wavenumbers 955 to 960 cm^−1^ and reaches the spectral position of 960 cm^−1^ at the end of the osteoid. At the mineralizing front of the osteoid, the peak is broader than in the growth zone and cortex. This indicates that the mineral begins to aggregate at the middle of the osteoid and undergoes some transformation across the osteoid and new bone tissues.

The shape of $\nu_3$ ${\text{PO}}_4^{3-}$ in the spectral region also varies with the distance of the osteoblast. At the end of the growth zone, the spectra in this spectral region present two small dominant peaks at 1029 and 1085 cm^−1^ and a third peak that begins to grow at 1113 cm^−1^, whereas in the deep cortex the spectra present two peaks at 1034 and 1115 cm^−1^ indicated by two vertical black lines in Fig. 2b. The shape variation is due to subband unequal growths under this spectral region.

The XST index presents a linear augmentation with the distance from inside the growth zone (at 13 μm) until the end of the osteoid (at 32 μm) and in the middle cortex (58–90 μm), indicating that the 1030-cm^−1^-subband grows faster than the one at cm^−1^ (Fig. 4a). In the nearest cortex, just after the osteoid and until the middle cortex (from 32 to 58 μm), XST index magnitude decreases linearly with the distance due to faster growth of the 1020-cm^−1^ subband than the one of 1030-cm^−1^. In the deep cortex, after 90 μm, XST index magnitude is constant indicating an identical variation of 1020 and 1030-cm^−1^-subband intensities in this area.

The CM-index increases from inside the growth zone at 13  μm to the end of the osteoid at 35 μm (Fig. 4a). CM-index value is constant in the near cortex (from 35 to 55 μm) and increases again in the middle cortex and forms a plateau in the deep cortex at 95 μm. This indicates that the subband at 1030 cm^−1^ increases faster than the subband at 1110 cm^−1^ inside the osteoid and middle cortex. In addition, the absorption variation of the 1030- and 1110-cm^−1^ subbands in the near cortex (from 35 to 55 μm) and in the deep cortex after 95 μm, vary with the same proportion.

The APS-index intensity in Fig. 4a undergoes exponential decay from inside the growth zone to the deep cortex (at 90 μm) where it tends to zero. This shows that the absorption intensity of the subband at 1096 cm^−1^ increases exponentially and more intensely compared to the one at 1115 cm^−1^.

The mineral-to-matrix ratio (MM index) is plotted in a blue-dashed line in Fig. 4a. It begins to increase linearly from inside the growth zone (at 13 μm) with the distance until the end of the mineralized osteoid (at 35 μm) and continues to increase in the near cortex (from 35 to 58 μm). In the middle cortex, the ascension undergoes an attenuation and tends to form a plateau in the deep cortex (at 90  μm).

In the $\nu_2$ $CO_3^{2-}$ and $\nu_3$ $CO_3^{2-}$ spectral regions, the variations in the crystal’s carbonate contents at different anatomic locations cause different absorption-subband variations (A- and B-type carbonate). The typical-observed-subband variations in the $\nu_2$ $CO_3^{2-}$ and $\nu_3$ $CO_3^{2-}$ spectral regions are represented as a function of the distance from the osteoblasts in Fig. 4a. The B-type-subband intensity increases exponentially from inside the growth zone and the adjacent cortex (i.e. from 20 to 43 μm). Thereafter, the B-type-subband augmentation undergoes attenuation with the distance in the middle cortex and tends to a plateau reached in the deep cortex (at 95  μm). The A-type carbonate-subband intensity follows the same variation as the B-type-carbonate-subband intensity. However, it reaches a plateau in the near cortex at 61 μm before the one of the B-type carbonate and at lower value than the B-type-carbonate subband as can be seen in Fig. 4a.

### Spatial Differences of Collagen and Phosphate Deposition

**Fig. 5:**
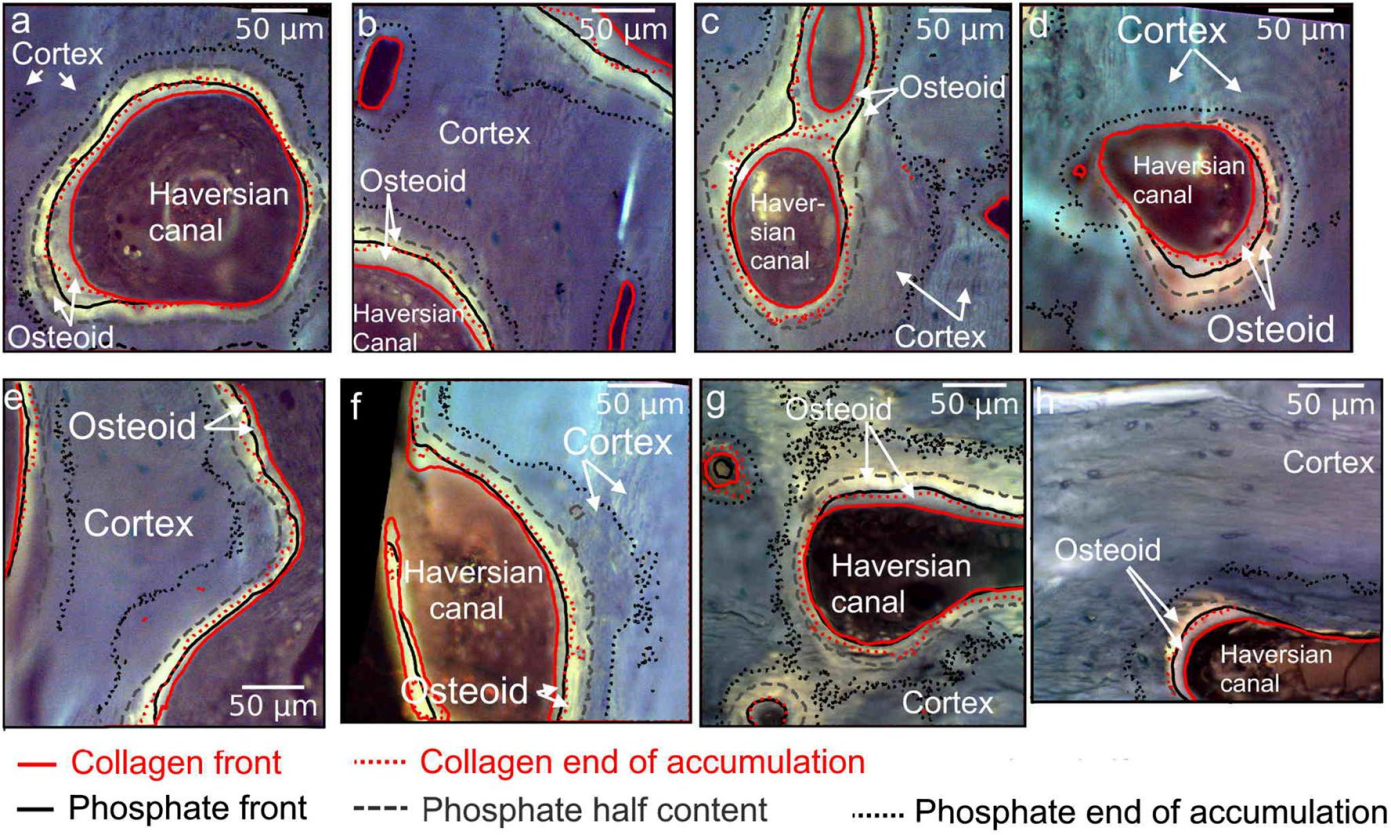
Collagen (at 1338 cm^−1^) and phosphate (at 960 cm^−1^) absorption-front lines represented in red and black respectively over the autofluorescence image of different osteons of different biopsies

The collagen- and mineral-aggregation fronts are presented in Fig. 5 in different osteons of different patients. In all osteons, the collagen begins to accumulate at the osteoblast layer before the phosphate apparition. The collagen content stops its linear augmentation at the middle of the osteoid, i.e. at the mineralizing front. This location corresponds almost to the location where the phosphate content begins to increase. The phosphate content stops increasing linearly in the new bone cortex. It should be noticed that the amount of phosphate reaches half of its final content at the end of the osteoid.

## Discussion

### New Bone Formation

New bone formation starts as osteoblasts begin the deposition of an organic matrix, 90% of which is constituted of type I collagen [16]. The collagen backbone consists of alpha chains of repetitive amino acid triplets that raise absorption subbands beneath amide I, II, III, A and B regions of the FTIR spectrum [9]. As expected, these different absorption bands are also observed in the absorption spectra of tissues in the entire osteoid and cortex (Fig. 2). In addition, the proline amino acid in collagen raises an absorption band in the FTIR spectrum at 1338 cm^−1^ which is specific to collagen type I [38]. The linear augmentation of the collagen-absorption band at 1338 cm^−1^ in the spectra of tissues inside the osteoid demonstrates a gradual accumulation of collagen. This deposition is mainly completed by the end of the growth zone. Next, the single alpha-chains fold into triple helices that are stabilized by hydrogen bonds provoking absorption in the infrared spectrum at the wavenumbers at around 3070 cm^−1^ , in the amide-B-spectral region [10]. In this study, this absorption band at 3070 cm^−1^, appears in the spectrum of tissues at the border with osteoblast layer and continues to increase for the tissues situated inside the osteoid with the tissue distance from the osteoblast border (Fig. 2), which indicates that the triple helix is consolidated immediately when the collagen is secreted.

The triple-helical collagen fibrils are then assembled into collagen fibers that are stabilized by the formation of covalent intermolecular cross-links. This phenomenon is called “collagen maturation”. First, three different immature-divalent cross-links are created: dehydro-hydro-lysinonor-leucine (deH-HLNL), dehydro-dihydro-lysinonor-leucine (deH-DHLNL), dehydro-lysinonor-leucine (deH-LNL) depending on the molecules involved in the linking [30]. In the mineralizing front and in the near cortex, because collagen accumulation is accomplished, the deH-DHLNL-subband-absorption diminution is due to the deH-DHLNL cross-link diminution only, which confirms the immature deH-DHLNL cross-links predominance in young bone [27].

Second, the immature-divalent cross-links are further assembled into mature trivalent cross-links: Hydroxylysyl-pyridinoline (Pyr), pyrroline (Prl), lysyl-pyridinoline (DPD) and deoxy-pyrrololine (d-Prl) [27, 31]. By consequence, immature deH-DHLNL cross-links should diminish for the profit of mature Pyr cross-link, which happens in the bone cortex of this study. Because the collagen accumulation is accomplished at the end of the osteoid, the absorption variation of Pyr-, DPD- and deH-DHLNL-subbands are proportional to the amount of created Pyr, DPD and reduced deH-DHLNL cross-links in cortex. In addition, the linear augmentation of XLR-index magnitude proves that deH-DHLNL cross-link is reduced in Pyr cross-link in the early cortex. Besides, the Pyr-subband-absorption variation observed in this study was also observed by Imbert et al. for cancellous vertebrae of sheep imaged with AFM-IR technique [20]. However, in the growth zone, Pyr-; DPD- and deH-DHLNL-subband intensity, and XLR-index magnitude variations are influenced by the collagen accumulation which is not completed in this region making it impossible to determine the absolute Pyr-quantity variation in this zone. Contrary to the study by Imbert et al. [20], who found that XLR-index magnitude is constant with the distance from the trabecular border, the XLR-index magnitude in this study increases in the cortex after the osteoid and then is constant in deep cortex. Our finding is in accordance with the study of human’s iliac crest bone by Faibish et al. [11]. This demonstrates a rapid collagen maturation in the cortex adjacent to the osteoid whereas no collagen maturation was observed in the deep cortex. The Pyr/DPD-index magnitude increases constantly in the early cortex until the deep cortex which indicates that Pyr-cross-link formation is higher than DPD-cross-link formation in bone as Viguet-Carrin et al. propose [42].

### Mineralization of Formed Collagen

During mineralization, the osteoblasts produce matrix vesicles transporting a precipitated constituent of calcium phosphate into the collagen scaffold [18, 25]. After its secretion, the calcium phosphates are transformed into amorphous calcium phosphates (ACP) which transform into octacalcium phosphate (OCP) and gradually into hydroxylapatite (HA) [41]. The phosphate in presence of crystalline fields provokes an absorption band in the infrared spectrum between 950 and 960 cm^−1^, i.e. in the $\nu_1$ ${\text{PO}}_4^{3-}$ spectral region. In this study, the mineral accumulation, represented by integrated intensity of the area under the $\nu_1$ ${\text{PO}}_4^{3-}$ region, presents a sigmoid variation with a strong slope in the mineralizing front and adjacent cortex to osteoid which indicates that the collagen mineralization appears only in mineralizing front and finishes further away in the cortex. The mineralizing front feature seems to be analogous to the mineralizing front observed in electron microscopy [25]. The linear augmentation of integrated value of the area under the $\nu _1$ ${\text{PO}}_4^{3-}$ region, demonstrates a progressive collagen mineralization in the mineralizing front of the osteoid and in the cortex adjacent to osteoid. In addition, at the end of the osteoid, the mineral content reaches 50% of its total amount indicating that the mineralizing front corresponds to the primary mineralization whereas the adjacent cortex corresponds to the secondary mineralization [2].

The transformation of ACP into HA provokes a progressive shift of the absorption band at 950 cm^−1^ to the right until it reaches 961 cm^−1^ [17, 36]. In this study, this absorption-band shift is observed for tissues inside the osteoid and continue to grow in the cortex adjacent to osteoid, which signifies a progressive formation of the HA, beginning slightly before the mineralizing front, and continuing to form in the mineralizing front and adjacent cortex. In addition, in osteoid, the evolution of the shape of the spectrum in the $\nu_3$ ${\text{PO}}_4^{3-}$ spectral region corresponds to the ones of autocatalytic conversion of amorphous calcium phosphate to poorly crystalline HA [32], which confirms the transformation into HA begining in osteoid. The formation of HA, in mineralizing front, is also observed by studying the absorption band due to $O-H$ at 3570 cm^−1^ because O–H-stretching vibration at this wavenumber is unique to crystalline hydroxylapatite [40]. In this study, the shoulder at around 3570 cm^−1^ is observed in mineralizing fronts which demonstrates that the formation of crystalline HA occurs in the mineralizing front of osteoid. This coincides with the beginning of the absorption band peak shifting observed in $\nu_1$ ${\text{PO}}_4^{3-}$ region

After hydroxyl-carbonate-apatite formation, mineral-maturity index (CM) starts to increase [44] i.e. the hydrated-surface layer covering the apatitic crystal, called non-apatitic environment, is progressively transformed into hydroxyl-carbonate apatite reducing the amount of non-apatitic ${\text{HPO}}_4^{2-}$ [43]. In this study, the variation of CM-index magnitude increases in the early cortex, and it reaches a plateau in the deep cortex indicating that bone-mineral maturation starts just after the osteoid when the HA is formed and continues to mature in the near cortex until the deep cortex. This variation is in accordance with studies about iliac crest bone in previous literature [12]. However, in the mineralizing fronts, CM-index magnitude is constant indicating that the mineral maturation may not begin yet. However, inside the osteoid and cortex adjacent to the osteoid, the mineral is still accumulating which creates non-apatitic ${\text{HPO}}_4^{2-}$ ions leading to an augmentation of the subband intensity at 1110 cm^−1^ and by consequence to a diminution of CM value preventing the use of CM index for any mineral-maturity characterization in osteoid.

Because the non-apatitic domains are transformed progressively in well-crystallized apatite, during the mineral maturation, the crystallinity should increase during this transformation. The crystallinity of bone mineral was proved to correlate with the variation of XST index, i.e. of the proportion of the subband intensity at 1030 cm^−1^ (subband found in well-ordered-crystallized material) relative to the one at 1020 cm^−1^ (subband found in poorly crystalline material) [33]. XST is a different characteristic than CM [12]. In this study, in the middle and deep cortex area (after 58  μm), XST index magnitudes increase first and then reache a plateau, indicating that crystallinity first increases and then remains constant in the deep cortex. This XST index variations correlate with the variations in cortex found for human iliac crest bone in the literature [7]. However the XST index describes the crystallinity of HA only if other components have not affected this index, which is the case in the middle and deep cortex where the transformation into HA and the phosphate accumulation is finished. This is not the case in osteoid and in its adjacent cortex. In these regions, the phosphate is still accumulating and undergoes several transformations leading to the creation of additional octacalcium and tetracalcium phosphate which will be transformed later into HA. These created calcium phosphates increase the absorption band at 1020 cm^−1^ of the XST index which could lead to the diminishion of XST observed in the cortex adjacent to osteoid whereas the crystallinity may not diminish [6].

During the maturation of the HA crystal several ion substitutions occur: substitution by a carbonate or by an acid phosphate [2]. In bone, predominant B-type-carbonate substitutions disturb the crystal shape and biomechanical properties [45] and increase the crystal solubility due to a weaker ${\text{Ca}}{-}{\text{CO}}_3$ bond than ${\text{Ca}}{-}{\text{PO}}_4$ bond [37]. Also, in this study, the amounts of B-type-carbonate substitute were superior to the A-type-carbonate substitute in the deep cortex. In the osteoid, the amounts of B- and A-type-carbonate substitutions increase slightly in the mineralizing fronts, demonstrating that carbonate substitution could already begin in osteoid. However, stronger substitutions are observed in the near cortex right after osteoid, indicating that the B- and A-type-carbonate substitutions occur mostly in the new cortex. In the deep cortex, both B- and A-type-subband intensities are almost constant. These results are in accordance with previous reports found in the literature [19, 28].

Acid phosphates (${\text{HPO}}_4^{2-}$) are found in bone apatite substituting a phosphate ion ${\text{PO}}_4^{3-}$ in HA [43]. In this study, APS-index magnitudes decrease strongly in the mineralizing fronts and in the early cortex indicating a stronger substitution by acid phosphate in these areas than in the deep cortex which confirms that the new-formed bones, presents a higher APS-index value than older bones [39].

In summary, it was demonstrated in this study that collagen aggregates in osteoid early before the mineralization initiation. The collagen aggregation is finished in the middle of osteoid, slightly after the mineralizing front. This temporal difference has also been referred to as mineralization lag time [22]. During collagen accumulation, collagen also starts to mature. However, the mineralization begins before than the collagen reaches its full maturity. The phosphates reach half of its final content by the end of the osteoid coinciding with the end of collagen accumulation and continue to accumulate in the cortex adjacent to osteoid until further away in the cortex. However, the mineral maturation appends right after the osteoid when the phosphate reaches its half content and ends further away in the deep cortex. When the phosphate begins its accumulation, it immediately undergoes carbonate and acid phosphate substitutions. The acid phosphate substitution diminishes as the crystal develops. In contrast, the amount of carbonate substitute increases with the accumulation of phosphate and ends in the deep cortex. These different variations and their relations presented in this paper confirm that mineralization and collagen deposition occur at different time points and that the mineralization begins when the collagen is not completely mature. This new information provides a better understanding of bone remodelling in human-mandibular bone which provides a base for studying bone pathologies caused by flaws in bone remodelling. In the future, differences in osteoid-mineralization patterns for non-healthy bones should be investigated to provide an explanation of the differences between bone types and pathologies.
